# On Better Estimating and Normalizing the Relationship between Clinical Parameters: Comparing Respiratory Modulations in the Photoplethysmogram and Blood Pressure Signal (DPOP versus PPV)

**DOI:** 10.1155/2015/576340

**Published:** 2015-01-27

**Authors:** Paul S. Addison, Rui Wang, Alberto A. Uribe, Sergio D. Bergese

**Affiliations:** ^1^Covidien Respiratory & Monitoring Solutions, Edinburgh EH26 0PJ, UK; ^2^Department of Anesthesiology, The Ohio State University Wexner Medical Center, Columbus, OH 43210, USA; ^3^Department of Neurological Surgery, The Ohio State University Wexner Medical Center, Columbus, OH 43210, USA

## Abstract

DPOP (ΔPOP or Delta-POP) is a noninvasive parameter which measures the strength of respiratory modulations present in the pulse oximeter waveform. It has been proposed as a noninvasive alternative to pulse pressure variation (PPV) used in the prediction of the response to volume expansion in hypovolemic patients. We considered a number of simple techniques for better determining the underlying relationship between the two parameters. It was shown numerically that baseline-induced signal errors were asymmetric in nature, which corresponded to observation, and we proposed a method which combines a least-median-of-squares estimator with the requirement that the relationship passes through the origin (the LMSO method). We further developed a method of normalization of the parameters through rescaling DPOP using the inverse gradient of the linear fitted relationship. We propose that this normalization method (LMSO-N) is applicable to the matching of a wide range of clinical parameters. It is also generally applicable to the self-normalizing of parameters whose behaviour may change slightly due to algorithmic improvements.

## 1. Introduction

**Figure 1:**
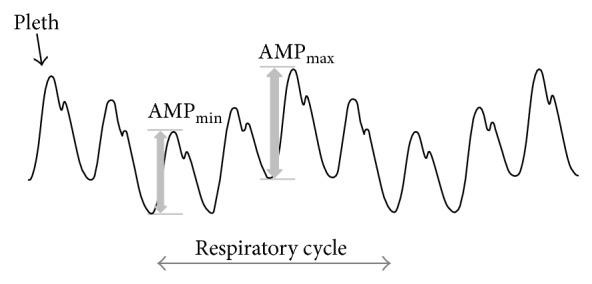
Deriving DPOP from the pleth. The amplitude of the cardiac component of the pleth waveform modulates due to respiratory-related changes in intrathoracic pressure which alters cardiac function. Principally, this stems from decreased left ventricular stroke volume during inspiration, leading to decreased pulse amplitude during this phase of respiration.

The respiratory modulation of the pulse oximetry photoplethysmograph (“*pleth*”) waveform is now a well-documented phenomenon. The modulation may manifest as amplitude, baseline, and/or frequency perturbations of the signal, depending on the physiological origin. Through sophisticated signal processing it has been shown that these perturbations may be used to determine* frequency* of respiration (respiratory rate) using commercially available hardware [1]. DPOP (ΔPOP or Delta-POP) is a noninvasive parameter which measures the* strength* of respiratory modulations present in the pleth waveform [2]. It is defined as [3]

(1) $$\begin{array}{c} \text{DPOP}=\frac{{\text{AMP}}_{\max}-{\text{AMP}}_{\min}}{\left({\text{AMP}}_{\max}+{\text{AMP}}_{\min}\right)/2}, \end{array}$$

where AMP_max⁡_ and AMP_min⁡_ are the maximum and minimum amplitudes of the cardiac pulse waveform in the pleth during a respiratory cycle. This is illustrated in Figure 1. DPOP has been proposed as a measure of the response to fluid administration in mechanically ventilated patients. As such it represents a noninvasive alternative for pulse pressure variation [4], a parameter derived from a blood pressure signal and widely used in practice for the prediction of the response to volume expansion in hypovolemic patients. PPV has the same mathematical formulation as DPOP [5] and many studies have shown favourable correlation between the two parameters [2, 6–12]. The role of PPV as a surrogate parameter for stroke volume variation (SVV) and its use in determining where on the Frank-Starling curve the patient is operating is not described here; rather the reader is referred to [2] by the author which contains an in-depth treatment of the subject. Reported studies often present the experimental data in terms of a scatter plot of DPOP against PPV with a line fitted to the data indicative of the relationship between the two parameters. In practice, this is often calculated using least-squares linear regression [13] which minimises the sum of the square of the residuals between the data and line of fit. However, this may not lead to the optimal estimate of the true underlying relationship between DPOP and PPV due to the asymmetric nature of noise present in the data. The aim of the present study was to examine methods to improve the determination of the underlying relationship between the two parameters.

## 2. Methods and Results

### 2.1. Patient Data and Data Acquisition Characteristics

With Institutional Review Board approval and written informed consent, a convenience sample of adult patients was enrolled at the Ohio State University Wexner Medical Center comprising mechanically ventilated patients requiring the placement of an intra-arterial line who had been scheduled for elective surgery. Each patient was fitted with a finger sensor (Nellcor OxiMax Max-A, Covidien, Boulder, CO). Synchronized acquisition of the pulse oximeter and arterial pressure signals was performed during the whole procedure and saved for later analysis. Further details of the acquisition system, patient exclusion criteria, and the data are provided in [14, 15]. The 20 subject data records used in the study had a mean length of data recording of 115 minutes (43–204 minutes). The data point pairs were decorrelated from each other by taking into account the IIR (infinite impulse response) filter characteristics and averaging buffer lengths in the algorithm. The algorithm outputs a new DPOP value every 5 seconds; this was subsampled to 180-second intervals to decorrelate the data to less than 1% shared information content between points. The oximeter device amplification on the pleth waveform was linear across the operating range and through gain changes. Full details of the algorithm used for DPOP are provided elsewhere by the authors [14, 15] (see also [1] and references contained therein for additional pertinent information on algorithms for the extraction of respiratory modulations for the derivation of respiratory information from a pleth).

### 2.2. On the Nature of the Data

**Figure 2:**
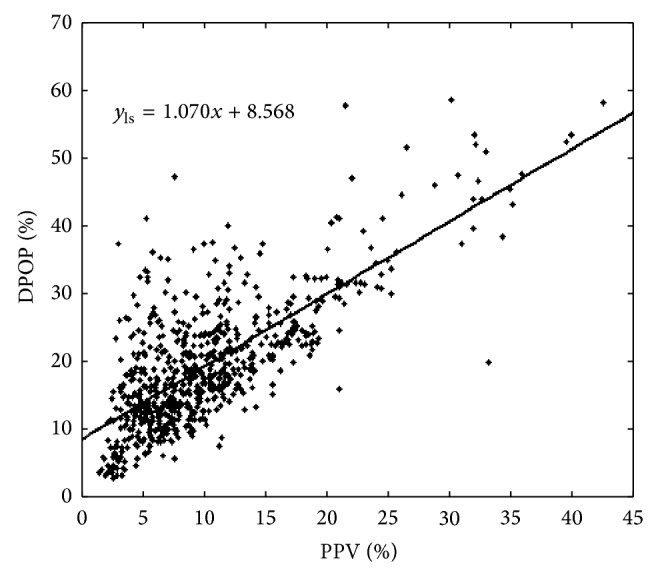
DPOP versus PPV data plot. Here the “best fit” line shown is calculated using the standard linear least-squares regression method.

An example of the least-squares fit for the DPOP-PPV data pairs is shown in Figure 2. However, we suspect that this is not the best estimate of the true relationship between DPOP and PPV as examples of tight linear DPOP-PPV relationships may be observed in the literature [3, 8, 10]. These other reported studies often involve manually selected data, where great lengths have been taken to ensure the best quality data. Figure 2, however, represents* all* data acquired during the clinical trial. This is more representative of the data that will be fed into a pulse oximeter in practice and hence contains significant noise. It is this kind of data we have a specific interest in within our development of robust parameters for use in medical devices. We also know that when there are no respiratory modulations present in the blood pressure (BP) waveform, there will be no respiratory modulations in the pulse oximeter waveform. Hence, assuming no other signal components, the relationship should pass through the origin. The regression line plotted in Figure 2 clearly does not do this, and the standard least-squares fitted line appears to be dragged upwards in the region of the origin due to the asymmetry in signal noise components.

**Figure 3:**
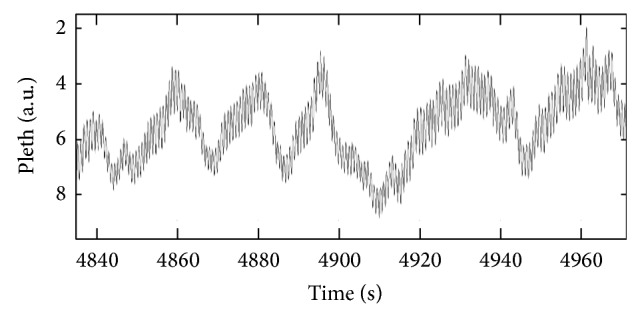
Example of a pulse oximeter waveform. The waveform from one of the patients in the study exhibits distinct long-term increases and decreases of the baseline.

**Figure 4:**
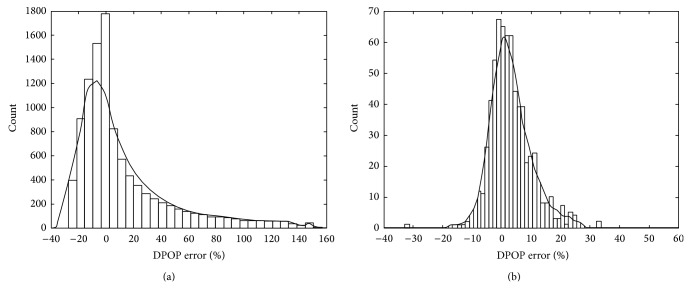
Distribution of gradient-associated errors in DPOP. A five-point smoothing distribution is overlaid on the histograms to aid interpretation. (a) Theoretical distribution using simple model with amplitude modulations ranging from 0 to *A*_min⁡_ and a gradient error from −*A*_min⁡_ to *A*_min⁡_. These are arbitrarily chosen ranges and other ranges lead to a similar positively skewed distribution of the error. (b) Actual distribution or errors from the LMSO method. Note that these will also include errors from other sources including random signal noise, although a positive skew is obvious in the data.

In an attempt to better understand the asymmetry in the distribution of noise in the DPOP measure, we examined more closely segments of signal where such errors occurred. Often they coincided with significant up slopes or down slopes in the baseline due to vasomotion. An example of this baseline slope is shown in Figure 3 where long-term (i.e., longer than the respiratory cycle) increases or decreases in the baseline are observed. We examined the effect of persistent gradients in the baseline on the calculated DPOP. A simple numerical model was developed whereby a uniformly occurring distribution of amplitude modulations ranging from 0 to *A*_min⁡_ and a range of baseline gradient induced amplitude measurement errors (as measured from the trough to pulse peak shown in Figure 1) from −*A*_min⁡_ to *A*_min⁡_ were input into (1). The plot of the resulting distribution of errors in DPOP is shown in Figure 4(a). The asymmetry is obvious in the plot. This may be compared to the actual distribution of DPOP errors from the least-median-of-squares line shown in Figure 4(b). There is a resemblance between the two figures with both possessing a distinctly positive skew. Although a very simple gradient induced noise model was used, the parametric bounds of the model can be varied quite widely (in terms of amplitude modulations and gradients) with the same conclusion: an asymmetric distribution with a positive skew results when gradient noise is introduced into the DPOP measure. Note that other noise will be present in the experimental data (motion, drug effects, etc.) and hence we do not expect an exact match with the synthetic data. In practice other noise sources could include those of a physiological nature (e.g., venous blood movement [16–18], autonomic modulation [19], motion artefact [10], and arrhythmia [19]) and device specific noise (e.g., amplification and filtering effects [20] or arterial line flushes [19]).

### 2.3. Regression Line Approximations of the DPOP-PPV Relationship

Given that the data set shown in Figure 2 is relatively noisy, exhibiting the asymmetry described above, the question is, how may we numerically approximate this relationship line better to account for the effects of the asymmetric spread of data points and thus improve the representation of the underlying association? We propose a number of steps in this regard.

**Figure 5:**
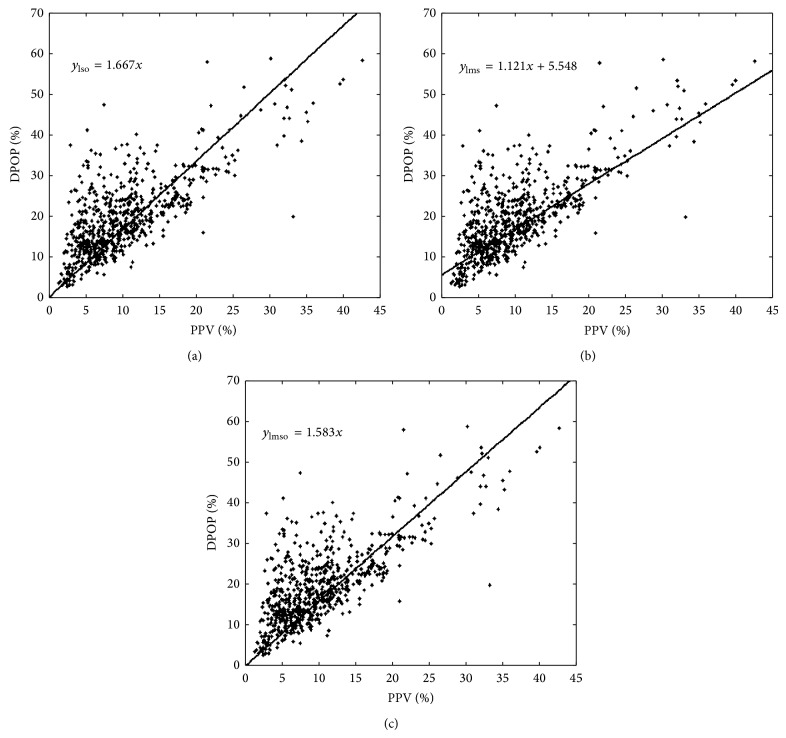
Alternative relationship line fits. (a) Least-squares forced through the origin: this method accounts for the relationship passing through the origin as expected from physiological considerations. (b) LMS method: this method is less susceptible to outliers, especially those which may skew the distribution. (c) LMSO method (LMS forced through origin): this combines the robust nature of the LMS method with the requirement for the relationship to pass through the origin.

First, we tackled the intent to have the fitted line pass through the origin. For least-squares linear regression this was actually a simple task. The mathematics of traditional least-squares regression was reworked with the extra criterion that the line intercept equalled zero [21]. This resulted in the modified regression line shown in Figure 5(a). We see that such an approximation appears to fit the original data better in the vicinity of the origin. However, as noted above, the noise manifests itself predominantly in terms of a positive error in DPOP which tends to drag the fitted line upwards from the suspected relationship. Many of these positive errors in DPOP are, in fact, quite large and thus dominate the least-squares technique. In an attempt to counteract the disproportional effect such outliers have on the traditional least-squares method we applied a least-median-of-squares (LMS) regression [22] to the data. The corresponding regression line for the data set is shown in Figure 5(b). We can see that this line appears to better approximate the suspected true relationship. Finally, we merged these two techniques, combining the desire to both account for outliers and pass through the origin.  Figure 5(c) plots an LMS regression line which has been forced to pass through the origin (the “LMSO” line).

### 2.4. Normalizing the Fluid Responsiveness Parameters

The steps described in the previous section were aimed at improving our estimate of the underlying relationship between the two parameters. However, in practice, either one or the other parameter is used to determine the fluid responsiveness of the patient (they are not normally used at the same time). The determination of fluid responsiveness is performed by comparing the parameter value against a predefined threshold, for example, 13% for PPV [3, 10, 19, 23, 24]. If the patient's PPV value is less than the threshold, then the patient may be deemed unresponsive to volume expansion and a decision was made not to administer fluid. Alternatively, if the patient's PPV value is greater than the threshold then this may indicate that the patient will respond to volume expansion. However, it is well known that this threshold is different for PPV and DPOP. This is also evidenced by the fact that these two parameters do not have 1 : 1 correspondence in the current data. This lack of parity is to be expected as the signals are quite different, with additional biomechanics at play between the BP generated pulse in the fluid (blood) column at the arm, where PPV is measured, and the pressure-mechanical coupling through the vessel walls and body tissue matrix “downstream” at the peripheral finger site where the pleth is measured [25, 26].

**Figure 6:**
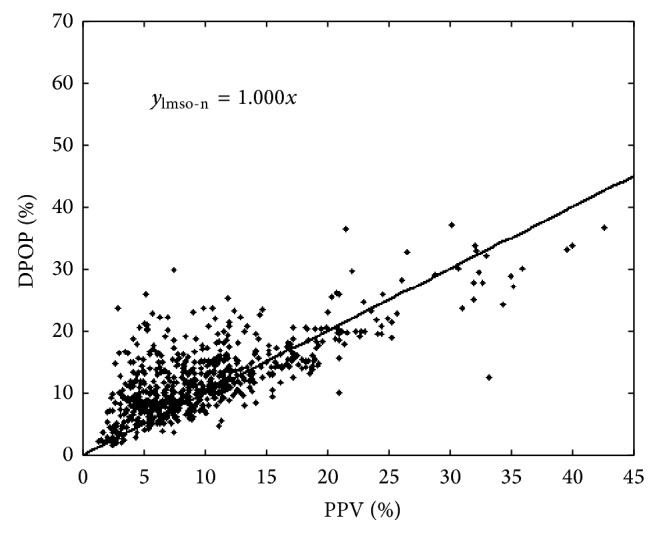
Relationship line normalization. By normalizing the relationship in this way, DPOP may be substituted directly for PPV as a fluid responsiveness parameter. In particular, the same threshold for indication of hypovolemia may be used for both parameters.

However, now that we have determined the linear relationship that passes through the origin (the LMSO method of Figure 5(c)), it is a trivial task to normalize the two measures. And, as we require DPOP to be the proxy for PPV, we rescale the DPOP values to match their corresponding PPV values. This is performed by multiplying each DPOP value by the inverse of the gradient of our best fit line. This rescaling, or normalizing, of the data using the LMSO line of Figure 5(c) is shown in Figure 6 (the “LMSO-N” method).

## 3. Conclusion

We have described a number of simple steps to improve the determination of the underlying relationship between two clinical parameters. These involved a least-median-of-squares estimator that is more robust to outlying data points and incorporating the additional criterion that the relationship must pass through the origin. The noise appeared asymmetric in nature and although we had no reference truth in this study, we know from the literature that tight linear DPOP-PPV relationships might be expected where great lengths have been taken to ensure the best quality data. If the DPOP parameter is being used to determine whether to administer fluids, then an error could possibly cause the wrong action to be taken (all medical monitoring devices exhibit some error and hence may be susceptible to this kind of issue to some degree). Whether a higher or lower DPOP value occurs at a threshold value of PPV (e.g., 13% [23]), indicative of the threshold for administering fluids, depends on the rotation and translation of the best fit line according to the regression and normalization used, which itself is data dependent. Further work on larger data sets is required to fully understand this effect. This area is ripe for more sophisticated analysis of this kind described here and the proposed method is perhaps a first step in this regard, although the authors do feel that it strikes a balance between improvement and oversophistication.

In addition, we have detailed a technique for normalizing the parameters using the inverse of the gradient of the best fit line. This method of normalizing DPOP in terms of PPV is advantageous in that the clinician may change between the two parameters seamlessly as there is no need for separate parameter-dependent thresholds. The LMSO-N method is also useful in self-normalizing any parameter against itself. For example, the method could be used to normalize the algorithm output of the parameter to the previous version of the same parameter, thus maintaining consistency across algorithm revisions.

Although we have discussed our methods in terms of the relationship between DPOP and PPV, other relationships both in terms of fluid responsiveness parameters (e.g., stroke volume variation and systolic pressure variation) and other medical parameters may benefit from this technique.
